# Assessment of exertional capacity using the six-minute step test in COVID-19 survivors six months after hospitalization: a prospective observational study in Paraíba, Brazil

**DOI:** 10.1016/j.bjid.2026.105787

**Published:** 2026-03-04

**Authors:** Vanessa L.A. Teotonio, Andre M. Siqueira, Giselle Duarte, Emanuelly Arruda, Ygor Soares, Alvaro Maciel, Eric L.A. Teotonio, José Moreira

**Affiliations:** a Universidade Federal da Paraíba (UFPB), Departament of Infectious, Inflammatory and Parasitic Diseases, João Pessoa, PA, Brazil; b Secretaria de Saúde do Estado da Paraíba (SESPB), Dr. Clementino Fraga Hospital Complex, João Pessoa, PA, Brazil; c Fundação Oswaldo Cruz (FIOCRUZ), Instituto Nacional de Infectologia Dr. Evandro Chagas (INI), Rio de Janeiro, RJ, Brazil; d Faculdade de Ciências Médicas da Paraíba ‒ Afya, Medicine Department, João Pessoa, PA, Brazil; e Universidade Federal do Rio Grande do Norte Federal (UFRN), Natal, RN, Brazil; f Instituto Butantan, São Paulo, SP, Brazil

**Keywords:** COVID-19, Survivors, 6MST, Step test, Exertional capacity

## Abstract

Survivors of Coronavirus Disease (COVID-19) frequently report persistent symptoms and reduced exercise capacity after hospital discharge. Simple, accessible methods to assess exertional performance in this population are urgently needed. We conducted a prospective observational study to evaluate exertional capacity using the six-Minute Step Test (6MST) in adults who had been hospitalized for COVID-19 at a referral center in Paraíba, Brazil. Participants were assessed six months after discharge using clinical evaluation, spirometry, standardized questionnaires (mMRC – modified Medical Research Council, SF-12 – Short Form-12), chest computed tomography and 6MST. The primary outcome was the number of steps completed on the 6MST. Performance was compared with normative values. Multivariable regression was used to identify predictors of poor exertional performance. Among 102 eligible participants, 88 (86.3%) completed the 6MST. The mean number of steps was 132.8 ± 30.9. 37.8% performed below the predicted threshold for healthy individuals; 10% fell below the 10th percentile. In the final multivariable model (R^2^_Ajdjusted_ = 0.39), older age, female sex, greater post-COVID-19 dyspnea (mMRC), and lower Mental Component Scores (MCS-12) were independently associated with lower step counts. The 6MST is a feasible tool for identifying exertional impairment in COVID-19 survivors. Reduced performance was common, particularly among older women and those with residual dyspnea or poorer mental health. These findings support the use of the 6MST to triage patients for post-COVID-19 rehabilitation.

## Introduction

Coronavirus Disease - 2019 (COVID-19), caused by the SARS-CoV-2, has resulted in a substantial number of survivors with persistent symptoms, including reduced exercise capacity and decreased quality of life.^1^, ^2^, ^3^ While Cardiopulmonary Exercise Testing (CPET) remains the gold standard for evaluating functional capacity, its complexity and limited availability restrict its use in post-COVID follow-up care.^4^

Field-based tests, such as the six-Minute Step Test (6MST), offer an attractive alternative for assessing exertional performance in resource-limited settings. The 6MST is easy to administer, requires minimal equipment, and correlates with aerobic capacity in chronic respiratory diseases.^5^, ^6^, ^7^ In contrast, the 6-Minute Walk Test (6MWT), considered the gold standard field test for evaluating chronic diseases requires a 30 m corridor to ensure reliable execution, as recommended by the American Thoracic Society.^8^^,^^9^ However, the 6MST applicability and performance characteristics in post-COVID-19 patients remain incompletely understood, especially in middle-income countries such as Brazil. And we hypothesized that 6MST would detect reduced exertional capacity in COVID-19 survivors, as the test has been validated in health people^10^ and in patients with chronic conditions.^5^, ^6^, ^7^ Furthermore, evidence has demonstrated correlations between the 6MWT, the sit-to-stand test, and the Chester Step Test in post-COVID-19 cohorts^11^ supporting the potential utility of step-based assessments in this population.

In this study, we aimed to evaluate exertional capacity using the 6MST in patients six months after hospitalization for COVID-19. We further explored demographic and clinical predictors of reduced exercise performance.

## Material and methods

This prospective observational cohort study included COVID-19 survivors, discharged from Dr. Clementino Fraga Reference Center for Infectious Diseases in Paraiba, Brazil.

Eligible participants included all patients aged 18-years or older who were discharged after hospitalization with RT-PCR-confirmed SARS-CoV-2 infection between May 1st and December 31st, 2020. Recruitment occurred between November 2020 and June 2021. Follow-up continued for 12-months, and data from the 6-month evaluation were analyzed.

The exclusion criteria consisted of patients who died before the follow-up visit, who declined to participate, who were unable to move freely to undergo in-person evaluation at the hospital or failure to establish contact by telephone. Eligible participants were contacted by telephone and invited to participate in the study approximately six months after hospital admission.

The study protocol was approved by the Research Ethics Committees of Paraiba State Health Department (number CAAE 39,928,920.8.0000.5186), and of the Oswaldo Cruz Foundation-FIOCRUZ (number CAAE 51,523,721.4.0000.5262).

The acute phase was defined as the interval between symptom onset and hospital discharge. Clinical data from the acute hospitalization phase were retrieved from medical records, following ISARIC forms, including demographic characteristics, clinical presentation, laboratory test results; information about Intensive Care Unit (ICU) admission, and requirement of Invasive Mechanical Ventilation (IMV). COVID-19 severity was classified according to the World Health Organization (WHO) criteria.^12^ The data were managed using REDCap software. All necessary measures were taken to ensure that the identity of the individuals studied was not disclosed and that no information could be linked to them, in accordance with the principles of confidentiality and privacy.

At the follow-up, during face-to-face interviews, the participants provided written informed consent, which was signed by the adult survivor or their legal representative. The survivors completed the modified British Medical Research Council (mMRC) dyspnea scale (scores 0‒4)^13^ and the 12-Item Short Form (SF-12) to assess quality of life (QoL). The mMRC score ≥ 1 indicates the presence of exertional dyspnea.^13^ The SF-12 reproduces both Physical Component Summary (PCS) and Mental Component Summary (MCS) scales, and scores below 50 in the American population indicate poor health-related quality of life.^14^

The clinical evaluation included anthropometric measurements, medical history, physical examination, pulmonary assessments, including pulmonary function; field exercise testing; and chest Computed Tomography (CT).

Spirometry was the pulmonary function test performed following American Thoracic Society (ATS)/European Respiratory Society (ERS) recommendations.^15^ The parameters recorded included Forced Vital Capacity (FVC), Forced Expiratory Volume in 1 s (FEV1), FEV1/FVC ratio expressed as percentages of predicted value. Abnormal results were considered below the lower limit of normal defined by equations for the Brazilian population.^16^

Chest CT images were assessed by expert radiologists. Residual abnormalities were considered as the presence of ground glass opacification, crazy-paving patterns, consolidation, linear opacities, or traction bronchiectasis.

The six-Minute Step Test (6MST) was used to assess functional exercise capacity. Participants were instructed to go up and down a 20 cm-high step without upper limb support and with verbal encouragement continuously for six minutes^10^ at the best possible speed, being allowed to slow down or stop as necessary, following the same recommendations of the 6-Minute Walk Test (6MWT) by ATS.^9^ The test was interrupted when subject’s request, exceeded 85 % of the predicted maximum heart rate, evidence of oxygen desaturation below 80 %, or any other sign that threatened the safety of the participant. The primary outcome was the total number of steps completed by the participant. Heart Rate (HR) and Peripheral Oxygen Saturation (SpO_2_) were measured at baseline, every minute during the test, and one minute after the test using a finger pulse oximeter. Oxygen desaturation was defined as a decrease in SpO_2_ of ≥4 % during the 6-Minute Step Test (6MST). Blood pressure and the dyspnea perception Borg scale were recorded at rest and immediately after the test. The patient’s predicted maximum heart rate (220 – age) was considered for submaximal test.^17^ Normative values for the number of steps climbed in the 6MST, by sex and age, as established for healthy Brazilian individuals, were used as a reference for our sample, and results below the normative values were considered as reduced functional exercise capacity, and the 10th percentile of the lower limit of normal was calculated within the sample to detect who had significant impairment exertional performance.^18^ Although ATS/ERS guidelines recommend duplicate field tests^19^ the 6MST was performed only once, in line with evidence suggesting that a single test may suffice post-COVID-19.^20^^,^^21^ The absence of pre-hospitalization data on pulmonary function, exercise capacity, or chest imaging hinders the interpretation of whether any abnormalities were pre-existing prior to COVID-19 infection. However, most published studies on post-COVID-19 pulmonary sequelae also lacked baseline data for comparison.

### Statistical analysis

Data were analyzed using Statistical Package for the Social Science (SPSS), version 20.0 (IBM Corp., Armonk, NY, USA). Due to the exploratory nature of the study during an emerging health crisis, a pre-determined power calculation was not feasible. Instead, we adopted a pragmatic, all-inclusive sampling strategy of eligible survivors within the study period. The number of steps climbed during 6MST was the outcome variable. The potential predictors and confounders were the clinical and epidemiological data during acute phase, and the results of spirometry, chest CT, mMRC dyspnea and SF-12 questionnaires during follow-up. Descriptive statistics were reported through table presenting categorical variables as absolute and relative frequencies. Normally and nonnormally distributed data were expressed as the mean and Standard Deviations (± SD) or median and Interquartile Range (IQR), respectively. Shapiro-Wilk test was used to determine the normality of variables. Variables with more than 20 % missing values were excluded from analysis.

The average number of steps climbed in 6MST was compared across categorical variables, using Student’s *t*-test or one-way ANOVA with Tukey post-test. Pearson’s correlation coefficient (*r*) was calculated to assess the association between the number of steps climbed in 6MST and independent continuous variables.

Multiple linear regression models were constructed to identify predictors of 6MST performance (number of steps climbed), with variables selected based on results from bivariate analyses. A significance level of α < 0.05 was applied in the final model and other tests, and 95 % Confidence Interval (95 % CI) was calculated.

## Results

**Fig. 1:**
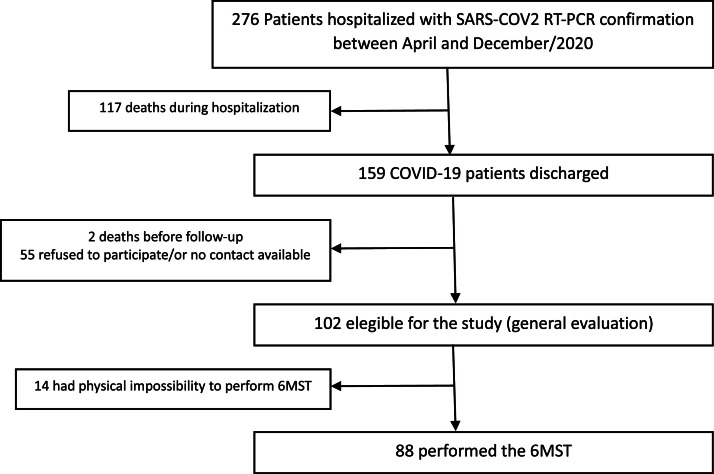
Study flowchart, showing COVID-19 survivors’ selection for this follow-up, after 6-months hospitalization.

Among the patients hospitalized during that period, 276 individuals presented RT-PCR-confirmed SARS-CoV-2 infection. 159 patients were discharged. Of these, 102 met eligibility criteria for follow-up, and 88 of 102 elegible participants completed the 6MST. Reasons for non-participation included inability to establish telephone contact, refusal to participate, and inability to move to the hospital for follow-up (Fig. 1)

The mean follow-up duration was 190-days. This sample comprised 64.7 % males, with a median age of 54-years (44‒64). The most prevalent comorbidities were hypertension (57.8 %), obesity (57.8 %) and diabetes mellitus (39.2 %). The median duration of hospitalization was 10-days (IQR 7‒14). Intensive Care Unit (ICU) was required in 38.2 % of cases, and 16.8 % needed invasive mechanical ventilation (Table 1).

**Table 1:** Baseline demographic and clinical characteristics of COVID-19 survivors during the hospitalization.

Variables	n/median/mean	%/IQR/±SD
**Demographics**		
Sex, n (%)		
Female	36	35.3
Male	66	64.7
Age median (IQR)	54	(44‒64)
BMI median (IQR) ‒ kg/m^2^	30.9	(28.8‒34.6)
**Comorbidities**		
Smoking, n (%)	43	42.6
Hypertension, n (%)	59	57.8
Cardiovascular disease, n (%)	9	8.9
Asthma, n (%)	6	5.9
COPD, n (%)	10	9.9
Obesity, n (%)	59	57.8
Diabetes, n (%)	40	39.2
**In-hospital characteristics**		
SOFA admission median (IQR)	2	(2‒3)
Lenght hospital stay (days) ‒ median (IQR)	10	(7‒14)
ICU, n (%)	39	38.2
IMV, n (%)	17	16.8
Severity COVID-19, n (%)		
Mild/Moderate	27	26.5
Severe/Critical	75	73.5

IQR, Interquartile Range; SD, Standard Deviation; BMI, Body Mass Index; COPD, Chronic Obstructive Pulmonary Disease; SOFA, Sequential Organ Failure Assessment; ICU, Intensive Care Unit; IMV, Invasive Mechanical Ventilation.

At the six-month follow-up, 56 % of participants self-reported exertional dyspnea (mMRC ≥ 1). Spirometry revealed that 38.1 % of individuals had a Forced Vital Capacity (FVC) under the Lower Limit of Normal (LLN). Quality of life assessment using SF-12 showed a median of Physical Component Score (PCS) below 50, indicating impaired physical health. Although none of the participants exhibited abnormal resting oxygen saturation, 10 % of patients experienced oxygen desaturation (defined as a ≥ 4 % drop in SpO_2_) during the 6MST. All individuals with exertional desaturation had comorbidities, one had asthma, three former smokers, seven with hypertension and obesity, one with chronic kidney disease and hypertension (Table 2).

**Table 2:** Clinical findings at follow-up of COVID-19 survivors after 6-months hospitalization.

Variables	n/median/mean	%/IQR/±SD
**Follow-up 6-months**		
Post COVID pain, n (%)	60	58.8
Post COVID weakness, n (%)	24	27
Post COVID cough, n (%)	17	17
Post COVID mMRC dypnoea, n (%)		
< 1	45	44
≥ 1	57	56
**Spirometry**		
FVC % mean (sd)	82.8	±12.8
FVC < LLN, n (%)	37	38.1
FEV1 % mean (SD)	84.6	±13.2
FEV1 < LLN, n (%)	34	35.1
FEV1/FVC mean (SD)	0.82	±0.05
FEV1/FVC < LLN, n (%)	10	10.3
**QoL SF-12**		
PCS median (IQR)	44.03	(37.67‒53.07)
MCS median (IQR)	52.82	(42‒57.9)
**Chest CT residual findings**		
Present, n (%)	52	51
**6min Step Test (6MST)**		
Number of steps climbed, mean (±SD)	132	±30.9
Number of steps below normative values, n (%)	34	37.8
Number of steps < 10th percentile, n (%)	9	10
SpO_2_ baseline (%), median (IQR)	97	97‒98
SpO_2_ nadir (%), median (IQR)	96	96‒98
Change in SpO_2_ ≥ 4 %, n (%)	9	10
HR baseline (beats/min), mean (±SD)	84.6	±13.5
HR (beats/min, %predicted), mean (±SD)	81.2	±10
Heart rate Post (beats/min), mean (±SD)	108.3	±16.63
Dyspnoea Borg pre, median (IQR)	0.0	0.0‒0.0
Dyspnoea Borg post, median (IQR)	7	6‒8

IQR, Interquartile Range; SD, Standard Deviation; mMRC, modified British Medical Research Council; FVC, Forced Vital Capacity; LLN, Lower Limit of Normality; FEV1, Forced Expiratory Volume in 1 s. QoL SF-12, Quality of Life Short Form-12; PCS, Physical Component Summary; MCS, Mental Component Summary; CT, Computed Tomography; SpO_2_, Peripheral Oxygen Saturation; HR, Heart Rate.

Participants climbed a mean of 132.8 ± 30.9 (95 % CI: 126.1–139.2) steps during the 6MST. When compared to established normative reference values for health Brazilian adults, 37.8 % of the participants demonstrated reduced functional exercise capacity. However, after calculating the 10th percentile cutoff derived from the same sample, impaired exertional capacity was identified in 10 % of participants (Table 2).

Bivariate analysis comparing the mean number of steps completed in the 6MST revealed statistically significant differences according to age, sex, smoking status, presence of hypertension, obesity; length of hospital stay; post-COVID-19 symptoms including pain, muscle weakness, exertional dyspnea. Additionally lower scores on the PCS-12 (Physical Component Summary) and MCS-12 (Mental Component Summary) of the SF-12 were associated with reduced 6MST performance. No statistically significant differences were found in relation to pulmonary function parameters in spirometry, ICU admission, IMV requirement, or residual abnormalities on CT images. (Tables 1, Table 2, Table 3 in the Supplementary Information).

**Table 3:** Predictors of number of steps climbed in 6MST among COVID-19 survivors (final multiple linear regression model).

Variables (final model)	Non-standard Coeficient		Standard Coeficient	p	95 % CI	
	Beta	Error-standard	Beta		Lower	Upper
**Constant**	158.785	16.0		<0.001	126.8	190.7
**Sex**	−22.153	5.4	−0.34	<0.001	−32.1	−11.3
**MCS**	.782	0.25	0.26	0.002	0.28	1.28
**Age (years)**	−1.036	0.20	−0.42	<0.001	−1.44	−0.62
**Post COVID mMRC dypnoea**	−17.086	7.5	−0.18	0.02	−32.1	−2.1

MCS, Mental Component Summary; mMRC, modified British Medical Research Council.

In the final multivariable linear regression model, female sex, higher post-COVID-19 dyspnea scores on the mMRC scale, lower scores on the Mental Component Summary (MCS-12) and old age remained as associated predictors with a lower number of steps completed in 6MST. The model explained 39 % of the variance in 6MST performance (R^2^_Adjusted_ = 0.39), suggesting that additional unmeasured factors contribute to exercise capacity in this population. In clinical exercise physiology, an explanatory power near 40 % is considered robust (Table 3).

## Discussion

In this prospective cohort of COVID-19 survivors assessed six months after hospital discharge, we found that a substantial proportion exhibited reduced exertional capacity as measured by the six-minute step test. Our findings underscore the clinical utility of the 6MST as a practical tool for identifying patients with residual functional impairment.

To our knowledge, there are few studies employing the 6MST to assess capacity in post-COVID-19 populations, particularly in middle-income settings. Similar to other post-COVID-19 cohorts, our sample was predominantly male, had a median age over 50-years^22^ and a median hospital length of stay of approximately 10-days, reflecting previously reported findings.^1^^,^^23^

In our cohort, the mean of steps climbed six-months after hospital discharge was 132.8 ± 30.9, which is markedly lower than the reference values for healthy Brazilian population (175 ± 45 steps).^18^ A prior study also found that healthy people climbed 34 steps more than post-COVID-19 group one month after discharged in the 6MST and walked approximately 94 meters more in the six-Minute Walk Test (6MWT).^24^ Similarly, another study using the Chester Step Test during 10-minutes, reported 150 steps climbed among COVID-19 survivors versus 250 steps in healthy controls.^11^ Among mechanically ventilated patients evaluated six weeks after discharge, a reduced mean of 86±39 climbed steps was documented.^25^

In bivariate analysis, variables such as age, sex, smoking, hypertension, obesity, post-COVID-19 symptoms (pain, weakness, dyspnea), and both the Physical (PCS-12) and Mental (MCS-12) components health-related quality of life were associated with the number of steps climbed in 6MST. However, the final multivariable model demonstrated that only female sex, older age, lower MCS-12 scores and higher mMRC dyspnea post-COVID-19 remained as associated predictors of lower performance. In the same line, obesity, age, sex and severity COVID-19 were considered strong predictors for reduced cardiorespiratory performance using CPET at post-COVID-19 follow-up^26^^,^^27^ the gold standard test for analyzing exertional capacity. While the exact mechanisms are not fully understood, factors like pre-existing health conditions, physical inactivity, pulmonary diffusion capacity, peripheral muscle strength, inflammatory and nutritional status and higher levels of depression influence physical performance. Previous studies have reported that women showed higher prevalence rates of anxiety and stress disorders than men, and these psychological conditions may adversely affect functional exercise capacity.^28^^,^^29^ This study did not assess cognitive impairment. Instead, health-related quality of life was evaluated using the 12-item short form health survey (SF-12). The Mental Component Summary (MCS) score of the SF-12 and female sex were identified as predictors associated with worse exertional capacity in the final multivariable model. The use of the adjusted coefficient ensures that this variance is not an artifact of model over-fitting but represents a parsimonious selection of clinically relevant predictors. Notably, the significance of the MCS-12 underscores that post-viral functional recovery is as much a psychological challenge as it is a physiological one.

Similarly, variables such as age, female sex, shorter height, higher body weight and cognitive impairment were contributing factors for reduced walking distance in 6MWT.^30^ And patients with persistent post-COVID-19 dyspnea exhibited reduced predicted percentage in 6MWT distance compared to non-dyspnea patients.^31^

Importantly, no significant differences in 6MST performance were found based on ICU admission, requirement for invasive mechanical ventilation, acute COVID-19 severity level, pulmonary function test results, or the presence of abnormal CT findings. These finds are aligned with other studies that have shown no correlation between walking test (6MWT) distances and severity of acute COVID-19 phase^32^ or between impaired pulmonary function and the lower exercise capacity in CPET.^33^

Exertional desaturation, defined as ≥4 % drop in Peripheral Oxygen Saturation (SpO_2_), is a clinically significant finding associated with worse prognosis in chronic lung diseases.^34^ Desaturation was observed in 10 % of participants during the test, and these participants presented comorbidities such as hypertension, obesity, asthma, smoking and chronic renal insufficiency. These results are consistent with previous findings showing desaturation during Cardiopulmonary Exercise Testing (CPET) in 8.6 % of individuals three months after hospital discharge for COVID-19.^33^ This phenomenon likely reflects the increased physiological demands of exercise, which require augmented oxygen delivery to skeletal muscles through enhanced cardiac output, ventilation, and pulmonary gas exchange, demands that are typically more pronounced in patients with comorbidities.^35^

The prevalence of reduced exertional performance in our study is consistent with broader literature on post-COVID-19 condition.^36^^,^^37^ The etiology of exercise limitation is multifactorial in this population, impairment can be related to circulation, ventilation, deconditioning or peripheral mechanisms. However, deconditioning was suggested as the leading cause of exercise limitation in post-COVID-19 condition, with some studies indicating a low occurrence of ventilatory limitation in CPET^33^^,^^38^ or no correlation between reduced peak exercise capacity and reduced diffusing capacity on lung function test or parenchymal lung disease on chest CT imaging.^39^ Notably, accurate investigation, that used invasive CPET in post-COVID-19 subjects, suggested a peripheral muscular, rather than a central cardiac limit to exercise.^40^

Persistent exertional dyspnea, reported by over half of the participants after 6-months, was also a significant predictor of reduced 6MST performance. That was consistent with evidence showing reduced V′O_2_ peak (maximum oxygen volume consumption) in dyspneic group during CPET.^39^ Even research in individuals with mild COVID-19 survivors, found dysfunctional breathing and hyperventilation pattern in this sample.^41^ Dyspnea is understood to result from physiological, psychological, social and environmental factors.^42^ further supporting our findings that dyspnea and impairment in the mental component of health-related quality of life along sex and age were predictors of the step test outcome.

This study has limitations. First, we employed a convenience sampling strategy without a priori sample size calculation, as the study was initiated when normative 6MST data for COVID-19 survivors were unavailable. While this may limit participant-to-predictor ratio, ensuring the stability of the adjusted R2. Second, the absence of a contemporaneous non-COVID-19 or non-hospitalized control group makes it challenging to differentiate the specific pathophysiology of COVID-19 from the general effects of post-hospitalization deconditioning or pre-existing comorbidities. This limitation should be interpreted since the study was conducted during the peak of the COVID-19 pandemic in Brazil, when the gamma variant predominated, a period that posed substantial logistical challenges to the recruitment of a healthy control group. Third, as a single-center study at a referral hospital, selection bias may favor more severe cases, potentially limiting generalizability to non-hospitalized populations. Finally, the lack of pre-infection functional data and the performance of only a single 6MST further necessitate caution in interpreting the absolute magnitude of functional decline.^19^ However, recent studies suggested that a single test may suffice, demonstrating stability between retests for assessing exercise capacity in COVID-19 survivors.^20^^,^^21^

Step tests have demonstrated validity in patients with respiratory diseases.^5^^,^^6^^,^^43^ During the COVID-19 pandemic, assessments of exercise capacity could not be performed in most centers. As a result, some pulmonary rehabilitation programs-initiated exercise testing in home-based settings, using tests with minimal cost and space requirements, such as step tests.^44^ The 6MST offers a pragmatic solution for the systematic integration of functional assessment into post-COVID-19 longitudinal care pathway, particularly in resource-constrained settings where access to 30-meter corridors for the 6MWT or equipment for CPET is limited. By requiring only a 20-cm step and a pulse oximeter, the test can be implemented at the primary care level to facilitate early triage.

We propose a clinical pathway where survivors are screened via the 6MST six-month post-discharge; those performing below the normative values, mainly if below the 10th percentile or demonstrating exertional desaturation (≥ 4 %) should be prioritized for multidisciplinary rehabilitation or targeted cardiovascular workup. This tiered approach ensures that intensive specialist resources are reserved for the most functionally impaired patients, while the 6MST´s sensitivity to psychological and symptomatic factors (MCS-12 and mMRC) allows for a truly holistic initial evaluation.

## Conclusions

The simplicity and minimal resource requirements of the 6MST make it an effective field-based alternative for the longitudinal assessment of exercise capacity in post–COVID-19 populations.

Exercise performance during the 6MST was frequently submaximal and significantly lower than normative values for healthy individuals, corroborating findings from other post–COVID-19 cohorts.

Routine implementation of the 6MST during follow-up may facilitate early identification of patients at risk of long-term physical impairment and help prioritize referrals to pulmonary rehabilitation programs.

The applicability of the 6MST is particularly relevant in resource-limited settings and for decentralized or home-based follow-up strategies, supporting its integration into post–COVID-19 care pathways.

## Authors’ contributions

VT, AS, GD, EA, YS, ET, JM: design and planning of the study. VT, EA, YS, ET: data collection. VT, AM: statistical analysis. VT, AS, AM, JM: interpretation of findings, writing and/or revision of all preliminar drafts; and VT, AS, GD, EA, YS, ET, JM: revision of the final version. All authors read and approved the final version.

## Funding

This research did not receive any specific grant from funding agencies in the public, commercial, or not-for-profit sectors.

## Data availability

The data that support the findings of this study are available from the corresponding author upon reasonable request.

## Conflicts of interest

The authors declare: no relationship with a person or organization that could affect one’s objectivity, or inappropriately influence this work. No funding source for this research.

## Appendix Supplementary materials

Image, application 1

## Footnotes

Supplementary material associated with this article can be found, in the online version, at doi:10.1016/j.bjid.2026.105787.
