# Supplementary material for: Assessment of exertional capacity using the six-minute step test in COVID-19 survivors six months after hospitalization: a prospective observational study in Paraíba, Brazil
Source: Braz J Infect Dis. 2026 Mar 4;30(2):105787. doi: 10.1016/j.bjid.2026.105787 (PMC12969291; doi:10.1016/j.bjid.2026.105787)
Supplement: Supplementary file 1 [file mmc1.docx]

BJID-D-25-00261

**SUPPLEMENTARY MATERIAL**

**Table 1** Comparison between average number of steps by sex and age group.

| **Age Group** | **Sex** | | | | | | **p-value** |
| --- | --- | --- | --- | --- | --- | --- | --- |
|  | **Male** | |  | **Female** | | |  |
|  | **Mean** | **±SD** | **Percentile 10** | **Mean** | **±SD** | **Percentile 10** |  |
| 20‒59 years old | 145.2 | 28.9 | 108.0 | 129.3 | 26.0 | 103.0 | 0.04 |
| Over 60-years old | 133.0 | 19.6 | 91.0 | 92.0 | 23.8 | 66.0 | <0.001 |
| Total | 142.8 | 26.7 | 99.0 | 113.7 | 29.9 | 84.00 | <0.001 |

**Table 2** Comparison between average number of steps in 6MST by categorical baseline characteristics and findings at follow-up.

| **Variables** | | **6MST number of steps climbed** | | **p-value** |
| --- | --- | --- | --- | --- |
|  |  | **Mean** | **±SD** |  |
| Smoking | Yes | 118.7 | 33.4 | 0.05 |
|  | No | 132.1 | 31.1 |  |
| Hypertension | Yes | 124.3 | 34.1 | 0.001 |
|  | No | 142.6 | 34.8 |  |
| Cardiovascular disease | Yes | 123.0 | 11.5 | 0.57 |
|  | No | 133.4 | 33.4 |  |
| Asthma | Yes | 136.0 | 30.8 | 0.41 |
|  | No | 132.6 | 33.4 |  |
| COPD | Yes | 130.4 | 31.4 | 0.4 |
|  | No | 133.8 | 33.4 |  |
| Obesity | Yes | 126.1 | 24.5 | 0.03 |
|  | No | 143.0 | 34.1 |  |
| Diabetes | Yes | 127.1 | 24.3 | 0.13 |
|  | No | 136.0 | 34.4 |  |
| ICU | Yes | 131.0 | 35.4 | 0.86 |
|  | No | 134.0 | 34.1 |  |
| IMV | Yes | 124.7 | 35.2 | 0.56 |
|  | No | 129.0 | 25.5 |  |
| COVID-19 Severity | Mild/Moderate | 135.3 | 33.8 |  |
|  | Severe/Critical | 131.9 | 30.1 | 0.66 |
| Post COVID pain | Yes | 126.0 | 24.4 | 0.02 |
|  | No | 141.8 | 34.5 |  |
| Post COVID weakness | Yes | 119.7 | 26.5 | 0.02 |
|  | No | 138.0 | 34.0 |  |
| Post COVID cough | Yes | 137.6 | 38.2 | 0.76 |
|  | No | 132.1 | 33.6 |  |
| Post COVID mMRC dypnoea | < 2 | 135.1 | 33.5 | 0.03 |
|  | ≥ 2 | 118.1 | 28.9 |  |
| Change in SpO_2_ ≥ 4% | Yes | 117.8 | 30.9 | 0.10 |
|  | No | 134.5 | 30.8 |  |
| FVC < LLN | Yes | 132.2 | 29.5 | 0.83 |
|  | No | 132.8 | 31.6 |  |
| FEV1 < LLN | Yes | 131.4 | 29.4 | 0.73 |
|  | No | 133.2 | 31.5 |  |
| FEV1/FVC < LLN (%) | Yes | 130 | 34.7 | 0.95 |
|  | No | 132.6 | 30.4 |  |
| PCS SF-12 < 50 | Yes | 124.3 | 31.2 | 0.002 |
|  | No | 144.9 | 27.1 |  |
| MCS SF-12 < 50 | Yes | 122.2 | 30.1 | 0.01 |
|  | No | 138.5 | 30.7 |  |
| CT residual findings | Present | 126.6 | 32.7 | 0.11 |
|  | Absent | 139 | 29.5 |  |

6MST, 6-Minute Step Test; SD, Standard Deviation; COPD, Chronic Obstructive Pulmonar Disease; ICU, Intensive Care Unit; IMV, Invasive Mechanical Ventilation; mMRC, Modified British Medical Research Council; SpO_2_, Peripheral Oxygen Saturation; FVC, Forced Vital Capacity; LLN, Lower Limit of Normality. FEV1, Forced Expiratory Volume in 1s. PCS, Physical Component Summary; MCS, Mental Component Summary; SF-12, Short Form-12; CT, Computed Tomography.

**Table 3** Correlation between number of steps climbed in 6MST and continuous variables by Pearson’s coefficient (*r*).

| **Variables** | **6MST- number of Steps** | |
| --- | --- | --- |
| Age | *r* | -0.41 |
|  | p | 0.001 |
| BMI | *r* | 0.05 |
|  | p | 0.60 |
| Lenght hospital stay | *r* | -0.27 |
|  | p | 0.008 |
| SOFA admission | *r* | -0.09 |
|  | p | 0.39 |
| SpO_2_ baseline (6MST) | *r* | -0.05 |
|  | p | 0.64 |
| SpO_2_ min SpO_2_ nadir (6MST) | *r* | -0.02 |
|  | p | 0.80 |
| HR (%predicted) (6MST) | *r* | 0.12 |
|  | p | 0.22 |
| PCS (SF-12) | *r* | 0.30 |
|  | p | 0.004 |
| MCS (SF-12) | *r* | 0.28 |
|  | p | 0.007 |

6MST, 6-minute step test; BMC, Body Mass Index; SOFA, Sequential Organ Failure Assessment; SpO_2_, Peripheral Oxygen Saturation; HR, Heart Rate; MCS, Mental Component Summary; PCS, Physical Component Summary; SF-12, Short Form-12.
